# Beyond Conventional Groper Appliance: A Modified Design for Dental Rehabilitation in Nephrotic Syndrome

**DOI:** 10.7759/cureus.113032

**Published:** 2026-07-20

**Authors:** N. J. Remya, Balagopal Varma R., Suresh Kumar J., Parvathy Kumaran, Arun Mamachan, Malini Venugopal, Nishna Thankappan

**Affiliations:** 1 Pediatric and Preventive Dentistry, Amrita School of Dentistry, Kochi, IND

**Keywords:** aesthetic rehabilitation, early childhood caries, functional rehabilitation, irreversible pulpitis, modified groper appliance, nephrotic syndrome, primary teeth

## Abstract

Groper appliance is a fixed esthetic space maintainer used to replace prematurely lost primary anterior teeth while supporting esthetics, oral function, and space maintenance. This case report describes the oral rehabilitation of a four-year-old girl with nephrotic syndrome in remission who presented with extensive dental caries affecting both the anterior and posterior primary dentition. The patient's medical condition was stable, and dental treatment was carried out in consultation with the pediatric nephrologist. Physician-directed medical precautions, including antibiotic coverage and corticosteroid supplementation, were implemented before invasive dental procedures to minimize potential complications. Clinical examination revealed chronic irreversible pulpitis and dentinal caries involving multiple teeth. Comprehensive dental management included pulp therapy, glass ionomer cement restorations, and the fabrication of a modified Groper appliance to replace the missing maxillary primary anterior teeth and compensate for the premature loss of the maxillary first primary molars. The appliance provided satisfactory esthetic rehabilitation with favorable short-term clinical outcomes, and both the patient and parents expressed satisfaction with the treatment. This case highlights the importance of early diagnosis, interdisciplinary management, and timely oral rehabilitation in medically compromised children and demonstrates the potential role of a modified Groper appliance as a practical treatment option in selected cases.

## Introduction

The consequences of early primary tooth loss, whether due to trauma or dental decay, include impaired chewing ability, decreased appearance, the possibility of developing abnormal oral habits, and trouble pronouncing labiodental sounds, significantly affecting the child’s behavior and psychological well-being [1].

In the early years, the anterior dentition plays an important role in the development of speech. Therefore, to restore all their functions, these teeth must be replaced. The Groper appliance is a suitable appliance for replacing anterior teeth [2].

Jasmin and Groper first documented the Groper appliance in 1984. The objectives were to enhance aesthetics, maintain space for the erupting permanent tooth, and support speech. Depending on the needs of the patient, it can be made in a variety of designs. Teeth were directly affixed to metal attachments, soldered onto a palatal bar in the previous design. Similarly, Shanmugaavel et al. developed the GRASCE appliance that substituted a stainless-steel crown for molar bands, and the artificial teeth were directly attached to metal components soldered to a palatal bar in earlier designs [3].

Nadelman et al. (2020) concluded that the premature loss of deciduous anterior teeth is associated with speech disturbances [4]. The speech difference is an important consideration for restoring anterior tooth loss because numerous sounds are phonated with the tongue contacting the lingual surface of maxillary anterior teeth [5].

Nephrotic syndrome is a nonspecific kidney disorder characterized by three characteristic features: proteinuria, hypoalbuminemia, and edema. Children with nephrotic syndrome have potential implications for long-term corticosteroid therapy, which can result in altered immune response and increased susceptibility to infection. It can also lead to uncontrolled bleeding from extraction wounds, delayed wound healing, and possible hypertension. A multidisciplinary collaboration, including communication with the child’s pediatrician/nephrologist, to evaluate disease status, medication history, and the need for appropriate precautions before undertaking dental treatment is inevitable. 

Oral manifestations are present in approximately 90% of patients with renal disease, as the condition as well as its treatment can affect the oral cavity and overall dental health. A reduction in erythropoietin production often results in anemia, which commonly presents as pallor of the oral mucosa. Gingival inflammation is also frequently observed and is primarily associated with plaque accumulation and inadequate oral hygiene. Extensive calculus formation can be frequently seen in children with chronic renal failure. There are altered salivary calcium (Ca), phosphorus (P), magnesium (Mg), oxalate (Ox), urea, and pH levels. The increased formation of calcium phosphate (CaP) and calcium oxalate (Ca-Ox) deposits leads to calculus deposition, which is mainly attributed to elevated salivary pH, along with reduced salivary magnesium levels and increased concentrations of salivary urea and phosphorus.

Dental care for children with nephrotic syndrome involves the proper prevention of oral diseases, as they have various factors that can contribute to the development of oral infections such as immunocompromised state, steroid therapy, poor healing capacity, increased calculus deposition, etc. Maintaining the teeth free of dental caries is truly important as they are at high risk of poor oral health, including gingival and periodontal health, which may be attributed to poor oral hygiene maintenance. Lifestyle differences, oral hygiene practices, and biological determinants such as differences in underlying susceptibility can all act as confounding factors [6].

The modified Groper appliance simultaneously restored anterior esthetics and function while maintaining the bilateral posterior spaces within a single fixed appliance. Although a conventional fixed anterior esthetic appliance could replace the missing anterior teeth, it would not preserve the posterior edentulous spaces, thereby requiring additional posterior space maintainers. Likewise, a removable prosthesis was deemed less suitable, as its effectiveness relies heavily on patient compliance, which is often unpredictable in children as young as four years of age. Furthermore, removable appliances are more susceptible to loss or breakage. A Nance appliance primarily serves as a space maintainer and anchorage device but does not replace missing anterior teeth or restore esthetics and speech. Alternatively, combining separate posterior space maintainers with a conventional anterior esthetic appliance would increase the complexity of treatment, prolong chairside time, raise costs, and require maintenance of multiple appliance components.

The main disadvantages of the Groper appliance are difficulty in maintaining proper oral hygiene, which increases the risk of decalcification of the remaining tooth, and food lodgment, thereby increasing the microbial load of the oral environment.

This case report aims to discuss the dental management of a patient diagnosed with nephrotic syndrome and systemic hypertension, having chronic irreversible pulpitis and deep dentinal caries on multiple teeth, in which prosthetic rehabilitation was done successfully by a modified Groper appliance, replacing anterior and posterior missing teeth.

## Case presentation

A four-year-old female patient was referred from the Department of Nephrology for the management of multiple decayed teeth. Medical history revealed nephrotic syndrome in remission with systemic hypertension. The patient was not on regular medication and attended routine medical follow-up every six months. Her blood pressure was well controlled, and she had been advised to follow a low-salt diet. As recommended by her physician, she was instructed to take a 2.5 mg dose of prednisolone before any anticipated stressful events and to receive antibiotic coverage prior to any invasive dental procedures. Frankl’s Behavior Rating Scale was Negative at her first appointment. The child had undergone the extraction of teeth 54 and 64 one year ago at a private clinic.

Oral examination revealed gross decay on teeth 51, 52, 53, 61, 62, and 63, with dental caries present on teeth 74, 75, and 84. Figure 1 presents a preoperative intraoral photograph of the maxillary arch showing root stumps of 51, 52, 61, 62, grossly decayed 53, 63, edentulous space of 54, 64. Figure 2 presents a preoperative intraoral photograph of the mandibular arch showing decayed 74, 75, and 84. Teeth 54 and 64 were extracted a year ago. There were redness and abscess in relation to teeth 52 and 62. Figure 3 shows the preoperative occlusion of the patient. Radiographic examination showed coronal radiolucency involving enamel, dentin, and pulp, indicating chronic irreversible pulpitis of teeth 51, 52, 53, 61, 62, and 63, with root resorption observed on teeth 51 and 61. Figure 4 shows the preoperative radiographic appearance of root stumps of 51, 52, 61, and 62. Teeth 74 and 75 exhibited coronal radiolucency involving enamel, dentin, and approaching pulp, suggestive of deep dentinal caries. Tooth 84 showed coronal radiolucency involving enamel and dentin, indicating dentinal caries.

**Figure 1:**
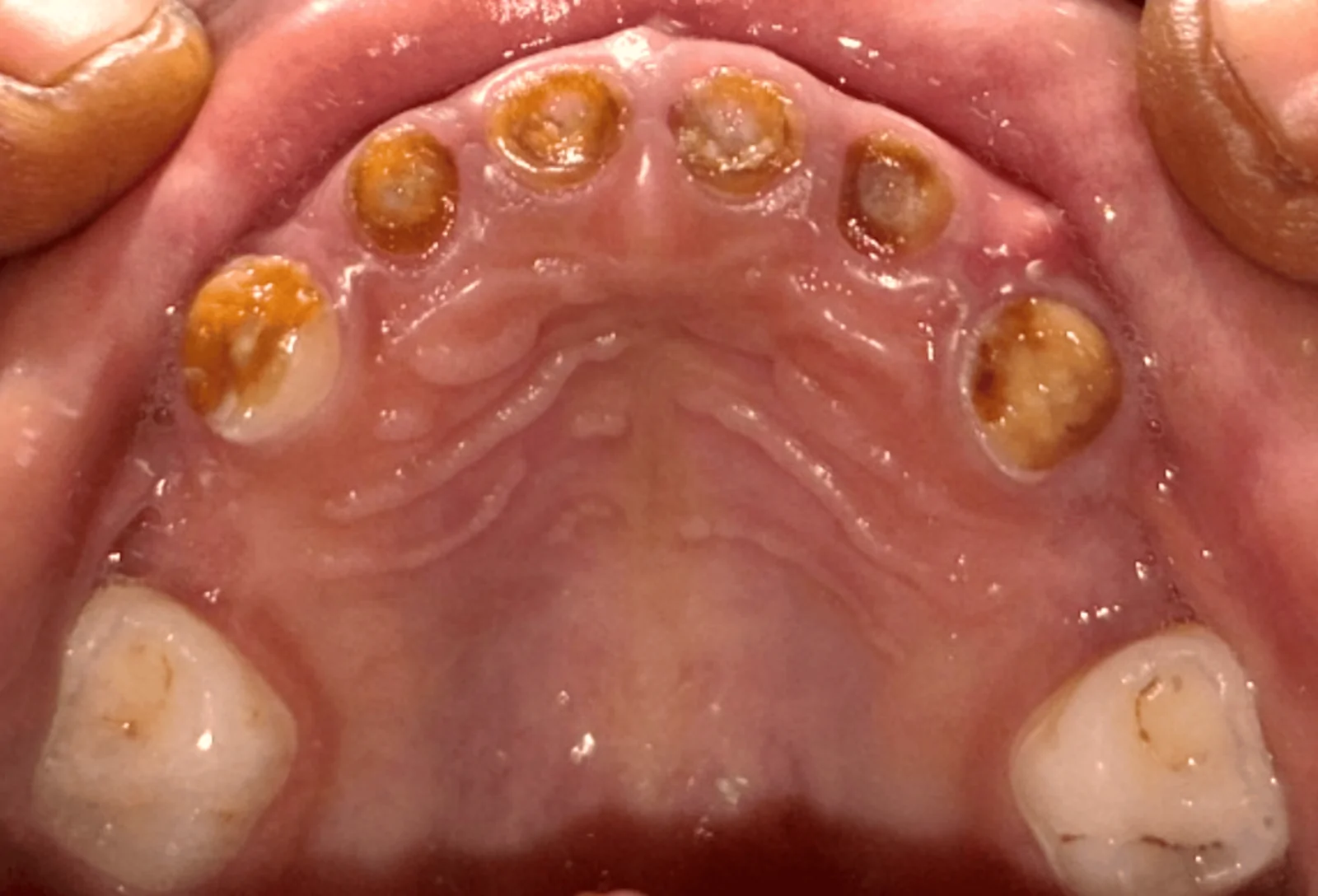
Preoperative intraoral picture of the maxillary arch showing root stumps of 51, 52, 61, and 62, grossly decayed 53 and 63, and edentulous space of 54 and 64

**Figure 2:**
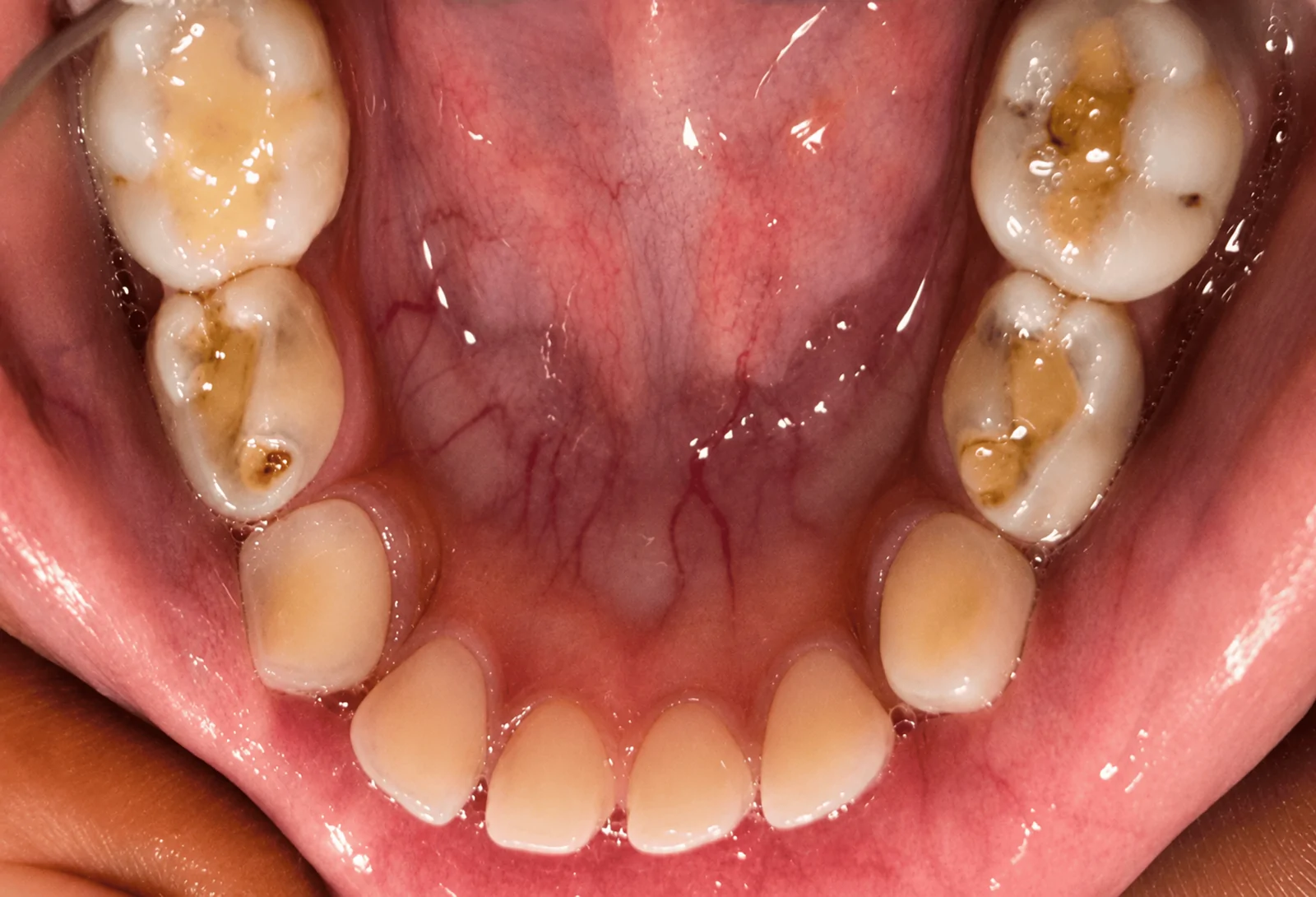
Preoperative intraoral picture of the mandibular arch showing decayed 74, 75, and 84

**Figure 3:**
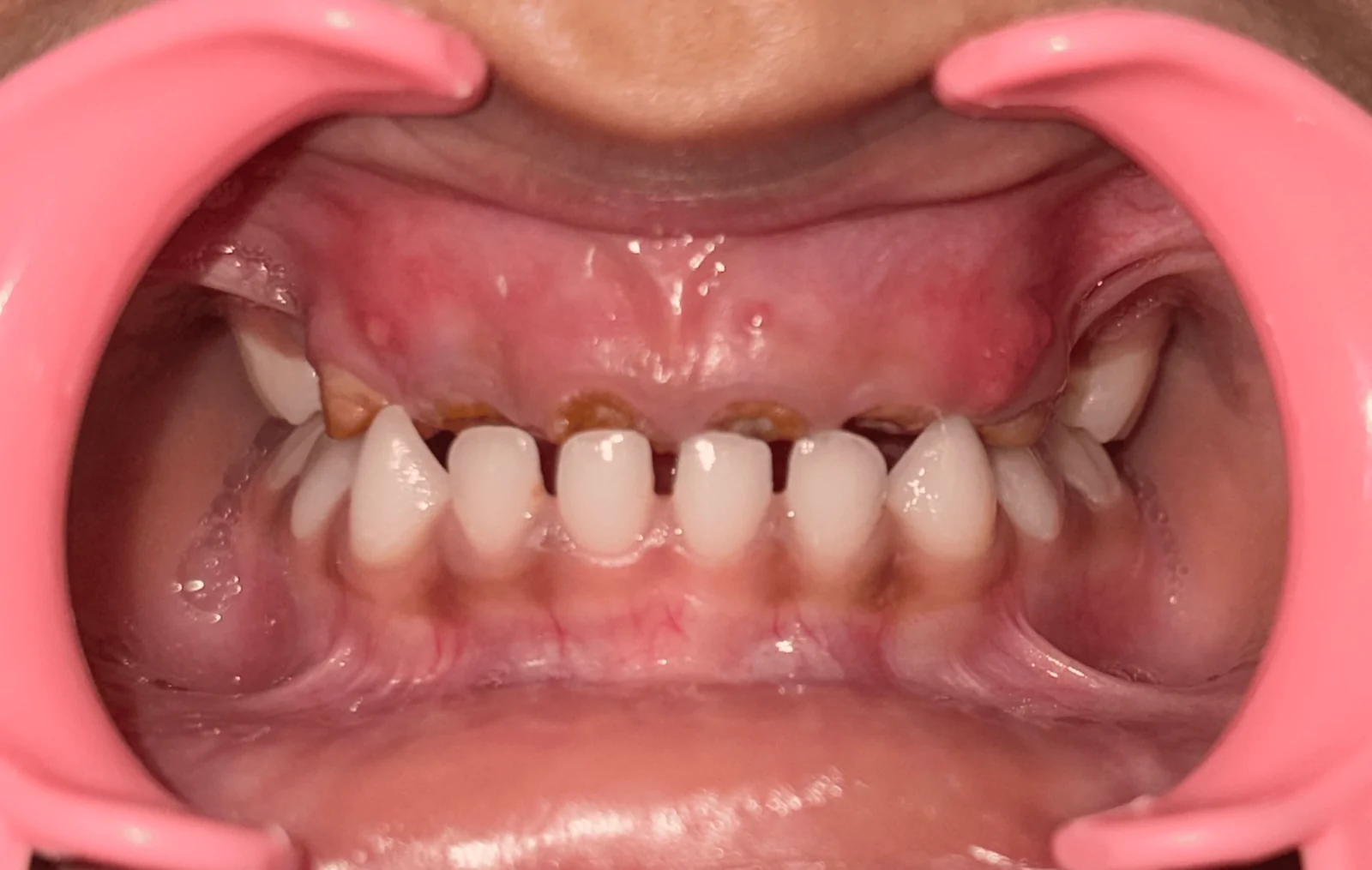
Preoperative occlusion of the patient

**Figure 4:**
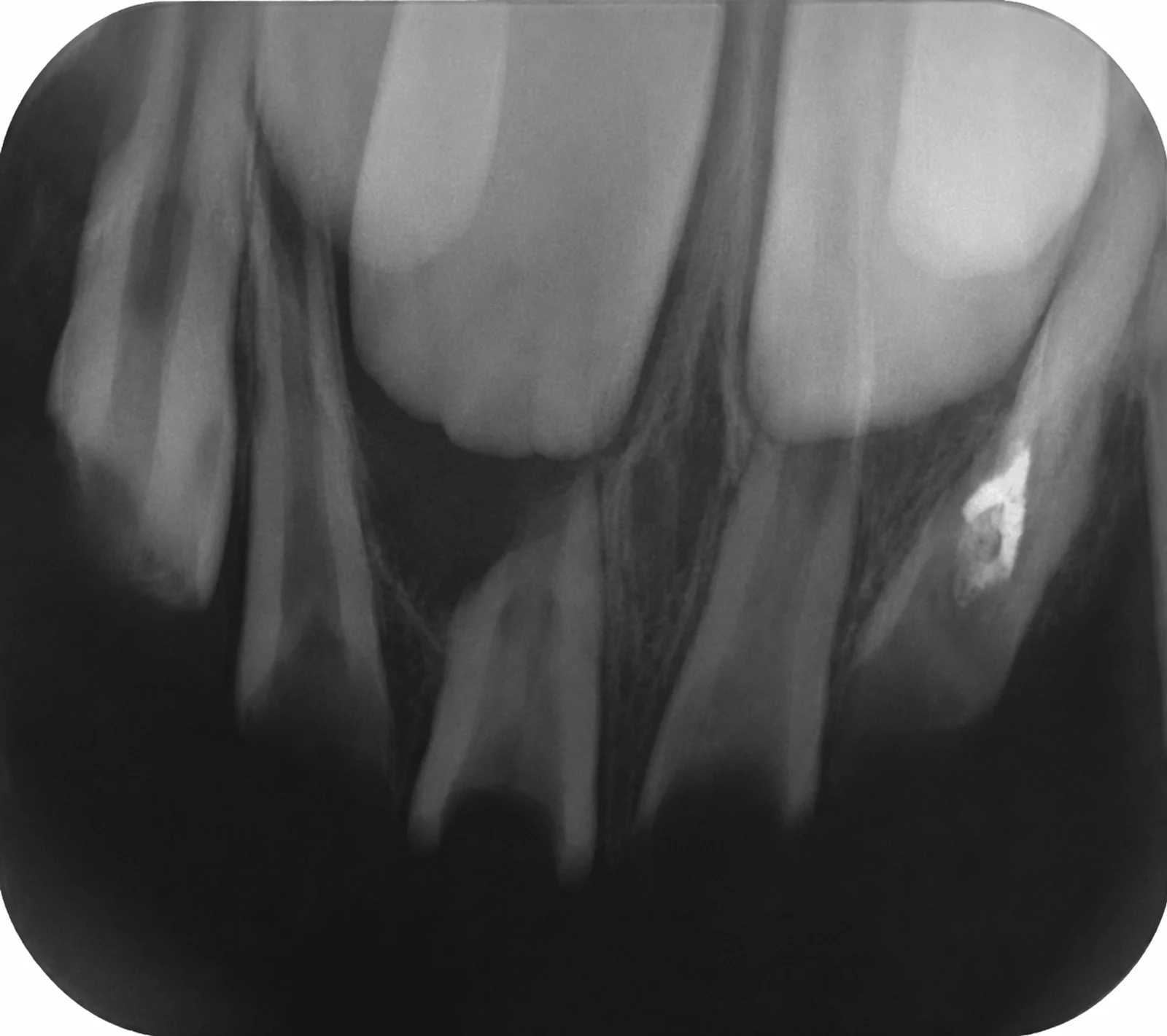
Preoperative radiographic appearance of root stumps of 51, 52, 61, and 62

According to oral and radiographic findings, the patient was diagnosed with chronic irreversible pulpitis on teeth 51, 52, 53, 61, 62, and 63, deep dentinal caries on teeth 74 and 75, and dentinal caries on tooth 84 with an edentulous space in relation to teeth 54 & 64. The dental therapy commenced after the pediatrician and nephrologist provided proper medical consent. Frankl’s behavior rating scale was negative.

The treatment was carried out in three phases. The preventive phase included diet counseling and oral hygiene instructions. The preparatory phase included indirect pulp capping on 74 and 75, pulpectomy of 53 and 63, and extraction of 51, 52, 61, and 62. Pulpectomy of 53 and 63 was planned even though it was grossly decayed, as primary canines exfoliate at 11 years of age, and maintaining them would act as a natural space maintainer.

The corrective phase included restoration of tooth 84 using Glass Ionomer Cement and a modified Groper appliance in relation to teeth 51, 52, 54, 61, 62, and 64 for aesthetic rehabilitation. Figure 5 shows the design of the modified Groper appliance used. The modification made in the appliance here is the addition of a single posterior tooth on either side for the replacement of the same, and to support mastication and occlusion. The maintenance phase included recall & review. The patient was advised to take the routine medications during the entire course of treatment.

**Figure 5:**
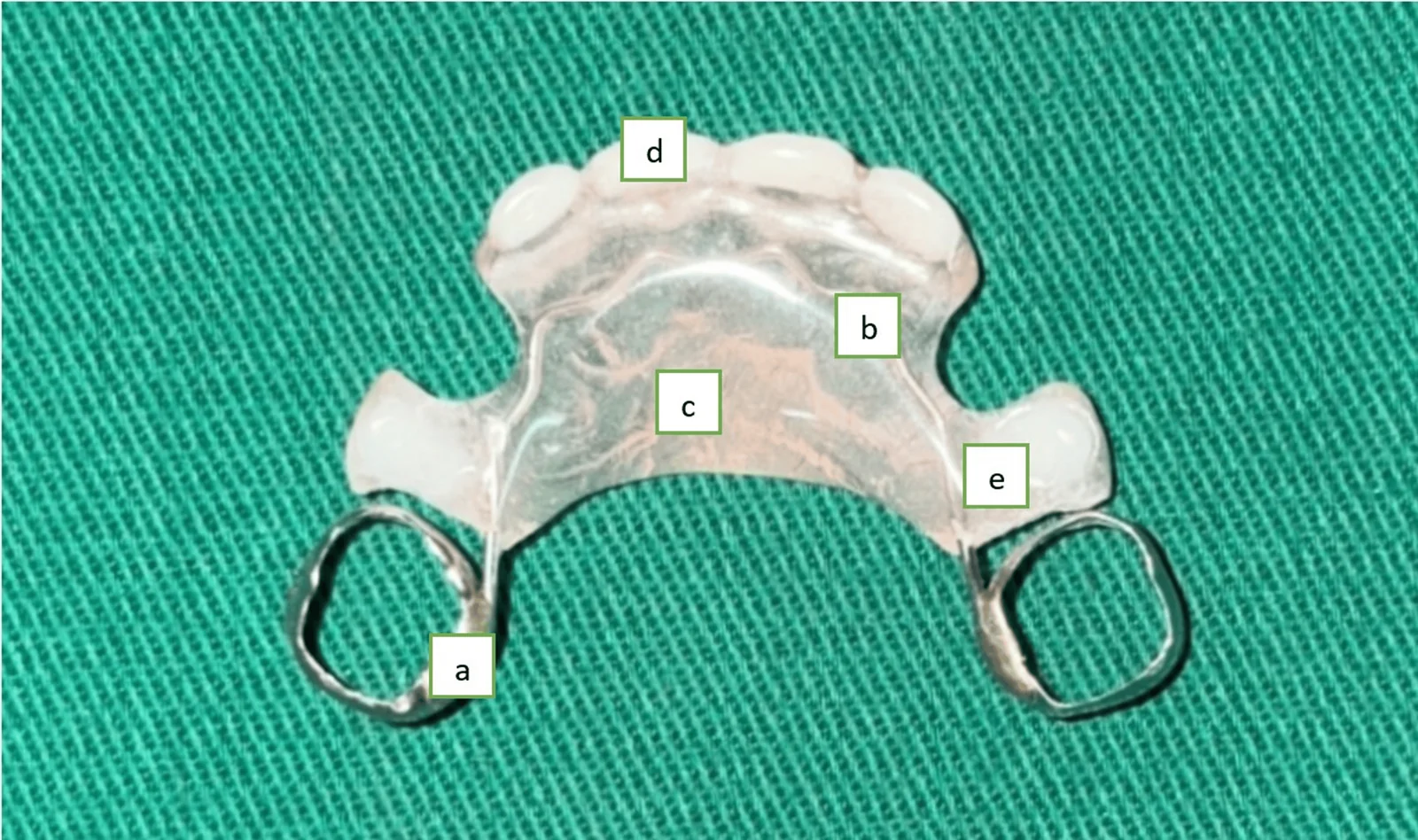
Design of the modified Groper appliance: (a) molar band, (b) wire framework, (c) acrylic plate, (d) anterior replacement teeth, and (e) posterior tooth extensions

During the first appointment, indirect pulp capping was performed on teeth 74 and 75, and glass ionomer cement restoration on tooth 84 to avoid any painful procedures at the first visit, as the child was apprehensive. A subsequent appointment was given after 3 days for pulpectomy of teeth 53 and 63. Two days before the appointment, the patient was advised to take amoxicillin 250mg/5ml, 3ml each thrice daily for five days as physician-directed coverage and metronidazole 200mg/5ml, 3ml each twice daily for three days for management of the existing abscess. She was also advised to take her regular dose of 2.5 mg of prednisolone, which she has been advised to take in case of any anticipated episode of stressful events.

The pulpectomy procedures were completed in two appointments to proceed to the next plan of treatment: the extraction of 51, 52, 61, and 62. Extractions were performed on the same day, but with much care and time to avoid chances of any uncontrolled bleeding that might present, as the patient is on prednisolone. After completion of extractions, the child was recalled to evaluate the status of healing.

After ensuring proper healing, the next step, the fabrication of the modified Groper appliance was initiated. Appropriately sized orthodontic molar bands were selected and adapted to teeth 55 and 65. An alginate impression of the maxillary arch was made with the bands in place, and the bands were stabilized within the impression before pouring the cast. An alginate impression of the mandibular arch was also obtained to prepare a working cast for establishing the occlusion during tooth arrangement. A framework was fabricated using a 1.0 mm round stainless steel wire extending between the maxillary molar bands, and the wire was soldered securely to the bands. Acrylic resin teeth were then selected, shaped to resemble the primary maxillary incisors, and adjusted to match the size and contour of the adjacent teeth.

A wax try-in was performed to verify the tooth arrangement, and there was no need for any occlusal adjustments. The child has been smiling with almost lost anterior teeth since a very young age. Thus, the trial fitting itself presented an abrupt change in her behavior from negative to positive, rated on Frankl’s behavior rating scale. Following this, a separating medium was applied to the cast, and the denture base was processed using cold-cure acrylic resin. The completed appliance was retrieved from the cast and evaluated intraorally to assess its fit and adaptation. After all necessary adjustments, the appliance was finished and polished before being cemented onto the teeth 55 and 65 using glass ionomer luting cement (GC Gold Label Type I, GC Corporation, Tokyo, Japan).

The modified appliance was the best choice as it simultaneously restored anterior esthetics and function while maintaining the bilateral posterior spaces within a single fixed appliance. A conventional fixed anterior esthetic appliance would have restored the missing anterior teeth but would not have maintained the posterior edentulous spaces, necessitating additional posterior space maintainers. Similarly, a removable prosthesis was considered less suitable because its success depends heavily on patient compliance, which can be unpredictable in children as young as a four-year-old, and it carries an increased risk of loss or breakage. A Nance appliance primarily provides anchorage and space maintenance but does not replace missing anterior teeth or restore esthetics and speech. Using separate posterior space maintainers together with a conventional anterior esthetic appliance would increase appliance complexity, chairside time, cost, and the number of components requiring maintenance.

The modified Groper appliance offered the advantage of combining anterior esthetic rehabilitation with bilateral posterior space maintenance in a single appliance, thereby simplifying treatment while improving patient comfort and acceptance. Nevertheless, like any fixed appliance, it has limitations, including the potential for plaque accumulation, gingival inflammation, band debonding, and the need for meticulous oral hygiene and periodic follow-up.

Figure 6 shows the postoperative occlusion of the patient, and Figure 7 shows the postoperative intraoral view of the maxillary arch after placement of the modified Groper appliance. The patient gained all the confidence and eagerness to smile, prompting her to take her own photographs. Figure 8 depicts the preoperative frontal smiling view of the patient, and Figure 9 depicts the postoperative frontal smiling view of the patient. Follow-up evaluations were conducted after two weeks, one month, three months, and six months to evaluate the post-treatment results. Postoperative reviews and evaluations demonstrated satisfactory appliance retention with stable bands and proper occlusion throughout the follow-up period.

**Figure 6:**
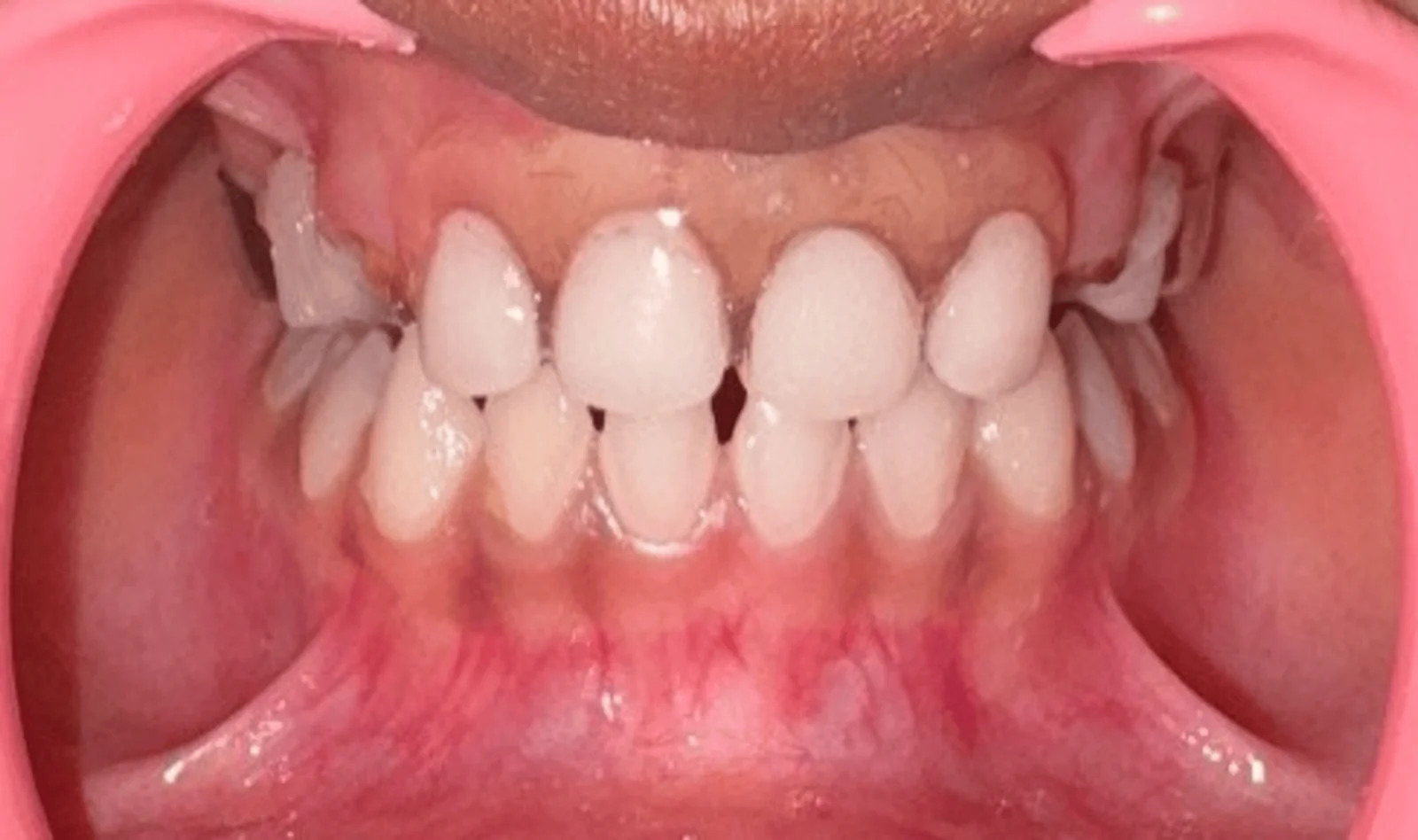
Postoperative occlusion of the patient

**Figure 7:**
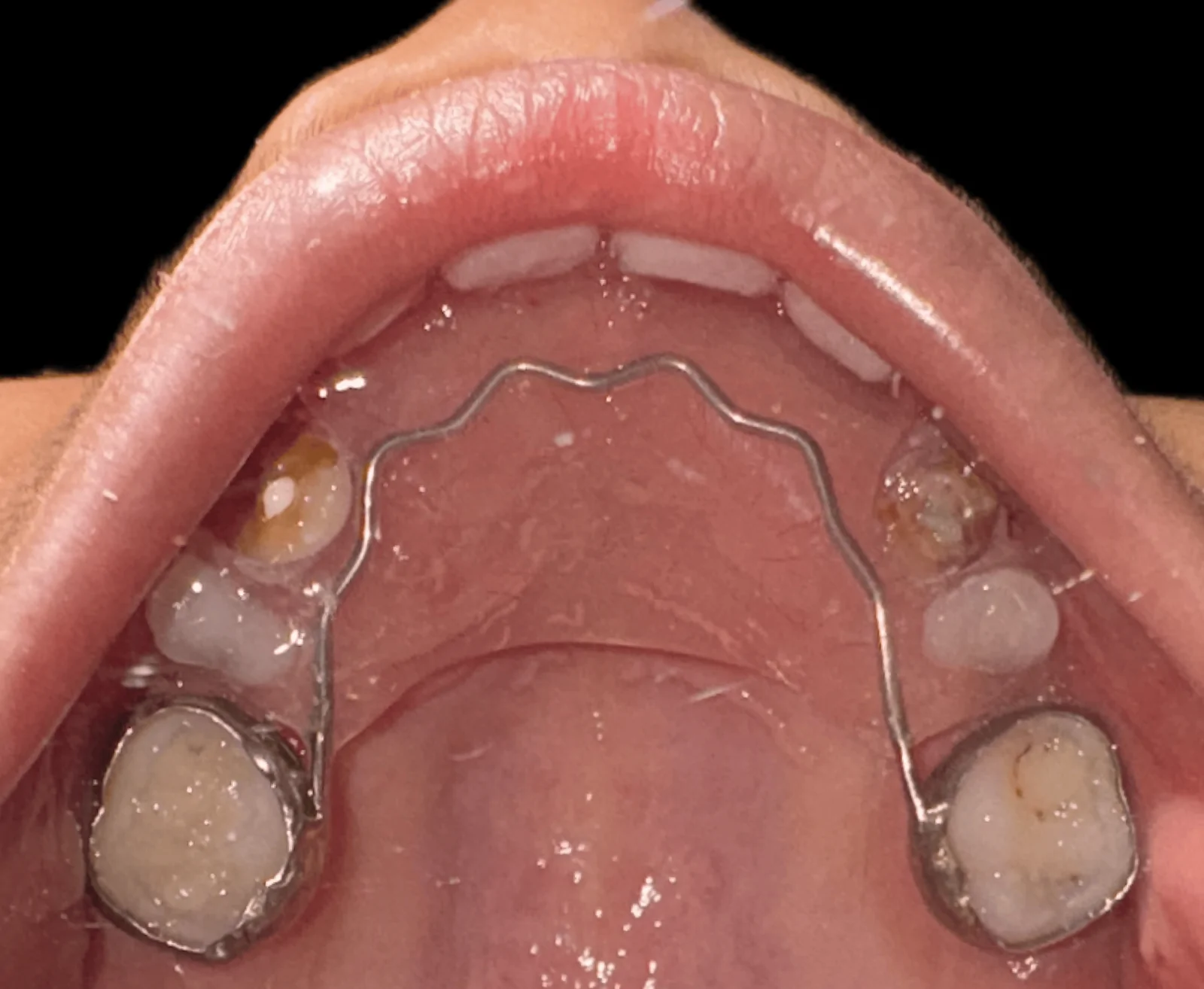
Postoperative intraoral view of the maxillary arch

**Figure 8:**
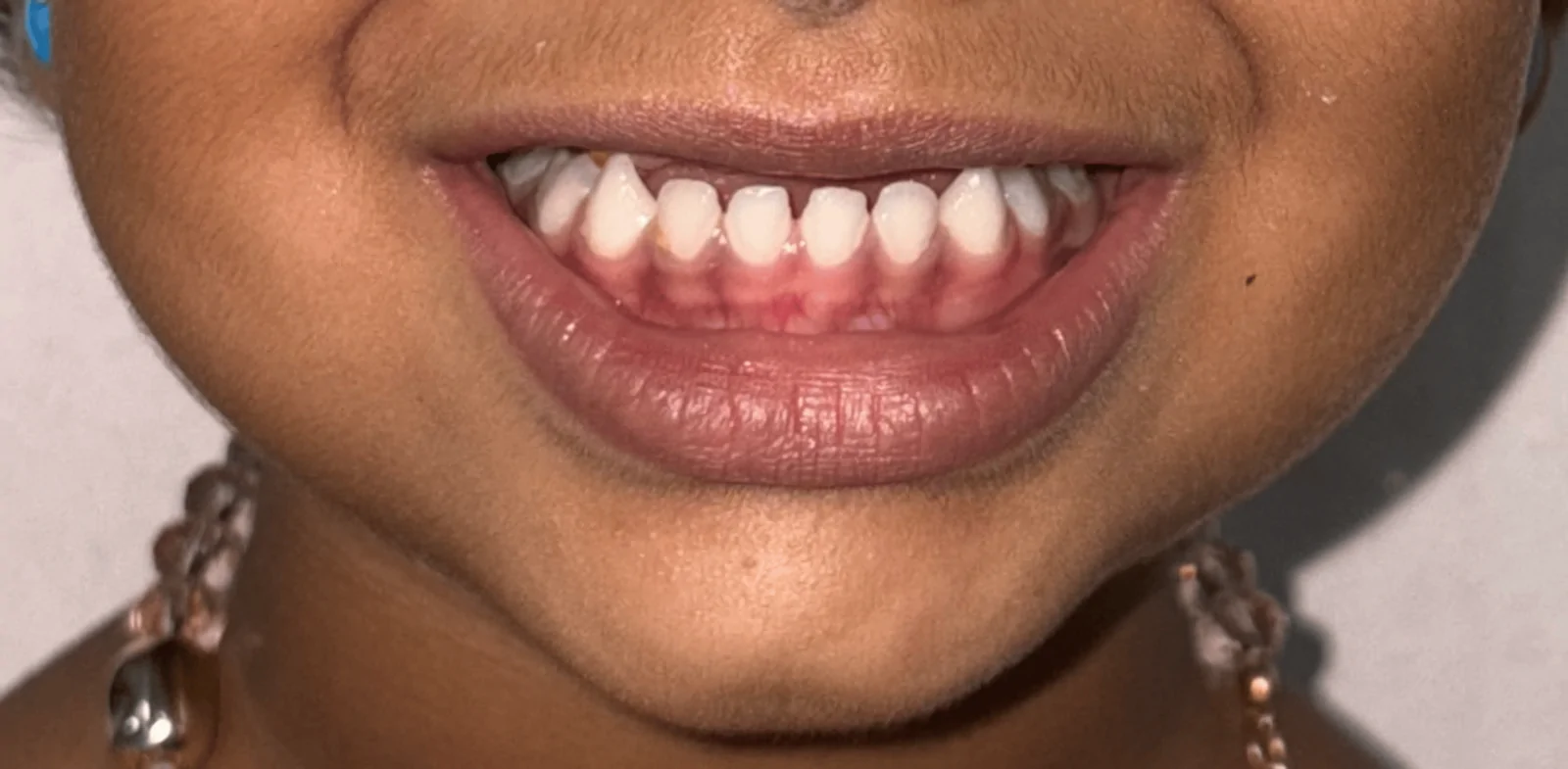
Preoperative frontal smiling photo

**Figure 9:**
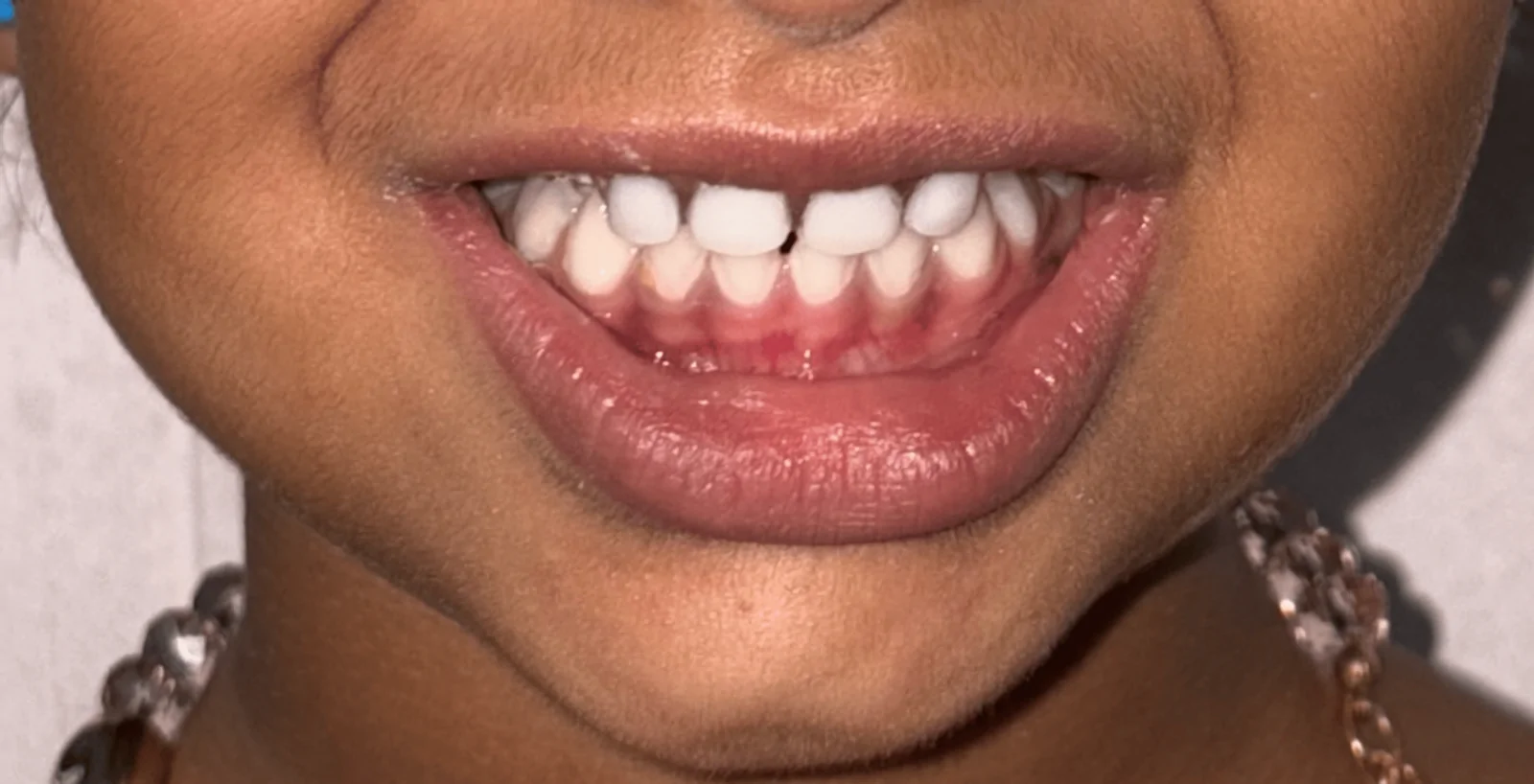
Postoperative frontal smiling photo

Oral hygiene was reinforced at each visit, and it was adequate. Gingival tissues remained healthy without evidence of inflammation; no recurrence of dental caries was noted; the appliance remained stable without debonding, and eruption of the permanent teeth was progressing as expected with satisfactory space maintenance. These objective clinical outcomes have been incorporated into the revised manuscript. Table 1 shows the summary of the appointments: treatment procedures, antibiotic coverage/medications, and follow-up findings. Proper care, including tooth brushing and proper rinsing, had been advised.

**Table 1:** Summary of appointments GIC: Glass ionomer cement

Sl. No.	Clinical Appointment	Treatment Procedures	Antibiotic Coverage/Medications	Follow-up Findings
1	Appointment for evaluation	Patient evaluation; fitness and medication advice sought from the pediatric nephrologist	—	—
2	First appointment	Indirect pulp capping on 74 and 75; GIC restoration of 84 to avoid painful procedures during the first visit	Regular dose of prednisolone 2.5 mg advised as prescribed for anticipated stressful events	No abnormal findings noted
3	Second appointment	Pulpectomy of 53 and 63	Two days before the appointment, the patient received amoxicillin 250 mg/5 mL, 3 mL three times daily for five days (physician-directed coverage), and metronidazole 200 mg/5 mL, 3 mL twice daily for three days for management of the existing abscess. Regular dose of prednisolone 2.5 mg was also taken as prescribed.	No abnormal findings noted
4	Third appointment	Extraction of 51, 52, 61, and 62	Same regimen as the second appointment	Good healing noted
5	Fourth appointment	Alginate impression recorded for fabrication of the modified Groper appliance	Same regimen as the first appointment	—
6	Appliance trial appointment	Trial of the appliance (molar bands and wax-up)	Same regimen as the first appointment	—
7	Fifth appointment	Delivery of the modified Groper appliance	Same regimen as the first appointment	No abnormal findings noted. Warm water rinses advised to prevent food impaction.
8	Sixth appointment	First review (two weeks)	—	Good compliance noted. The patient appeared happier and more confident and exhibited a positive dental attitude with excellent cooperation.
9	Seventh appointment	Second review (one month)	—	Good compliance noted. Oral hygiene maintenance was good.
10	Eighth appointment	Third review (three months)	—	Good compliance noted. Oral hygiene maintenance was good.
11	Ninth appointment	Fourth review (sjx months)	—	Good compliance noted. Oral hygiene maintenance remained good. The patient was preparing to travel abroad to stay with her parents.

## Discussion

The Groper appliance is conceptually similar to the Nance palatal arch appliance, consisting of acrylic teeth attached to a wire framework with an extension into the palatal region. The framework typically utilizes a round wire of approximately 0.036-0.040 inches in diameter, soldered to prefabricated stainless steel bands on the primary molars. Shorter wire spans are generally preferred, as they minimize interference with the eruption of permanent first molars, making first primary molars more favorable abutments than second molars [7].

In the present case, a modified Groper appliance was designed to address both anterior and posterior tooth loss. Acrylic teeth replacing the maxillary central and lateral incisors, along with the first primary molars bilaterally, were incorporated into a single acrylic palatal plate. The appliance was stabilized using a 0.036-inch round wire embedded within the acrylic and attached to bands on the primary second molars, thereby providing adequate retention and stability while restoring function and esthetics.

Various modifications of the Groper appliance have been reported in the literature to enhance its clinical applicability. Shanmugaavel et al. (2016) introduced the GRASCE appliance, wherein the wire arm was directly soldered onto stainless-steel crowns placed after pulpectomy, improving anchorage and durability [3]. Arikan et al. (2020) described a modification that allowed for transverse growth of the maxillary arch by incorporating a sliding mechanism using dual stainless-steel wires, facilitating eruption of permanent incisors [8]. Shrivastava et al. (2021) proposed a design incorporating brackets and a wire framework with buccal acrylic extension, enhancing retention and adaptability [9]. Joshi et al. (2021) further refined the appliance by adding a distal stop to prevent canine rotation or migration, thereby preserving arch integrity [10]. More recently, Saini et al. (2024) developed a dual-wire system with a crib-like configuration to improve composite retention and esthetics in anterior rehabilitation [1]. These variations highlight the versatility of the Groper appliance and emphasize the need for customization based on individual clinical scenarios. In contrast to previously reported designs, the present modification extends its utility beyond anterior replacement by simultaneously addressing posterior space loss, thereby providing a more comprehensive rehabilitative approach in a single appliance.

Although the literature does not conclusively establish that premature loss of maxillary incisors adversely affects arch development, their replacement is often indicated for functional, psychological, and social reasons. Children with missing anterior teeth may experience compromised esthetics and be subject to social discomfort or peer ridicule, which can affect their self-esteem [7]. Jabin et al. also reported that restoration of anterior esthetics using such appliances provides a significant psychological benefit to both the child and parents [11].

Furthermore, early loss of anterior teeth may contribute to speech difficulties, altered tongue posture, masticatory inefficiency, supra-eruption of opposing teeth, and undesirable tooth movements such as tipping and midline deviation [12]. Despite the lack of strong evidence supporting mandatory replacement in all cases, clinical decision-making should consider these potential consequences, as well as parental expectations, which often play a decisive role [7].

Conversely, some authors suggest that intervention may not always be necessary unless there is evidence of space loss, functional impairment, or development of deleterious oral habits such as tongue thrusting that could predispose to malocclusion [13]. Therefore, the decision to intervene should be individualized, balancing clinical findings with psychosocial considerations.

Management of pediatric patients with nephrotic syndrome requires careful medical evaluation and interdisciplinary coordination. Prophylactic antibiotic coverage is often not recommended for all children with nephrotic syndrome before dental procedures; instead, the decision should be individualized based on factors such as the patient's immunosuppressive status, presence of active infection, renal function, overall medical condition, and the invasiveness of the planned dental procedure, determined in consultation with the treating physician [14]. In the present case, antibiotics had already been initiated two days prior to the procedure after consultation with the concerned pediatric nephrologist, and therefore the conventional one-hour preoperative regimen was not followed.

In this case, both the patient and parents reported high levels of satisfaction with the functional and esthetic outcome, and compliance with follow-up visits was satisfactory. Despite its advantages, the appliance is not without limitations. Accumulation of plaque and food debris around the appliance can predispose to gingival inflammation and secondary caries [15]. Therefore, strict oral hygiene maintenance and regular follow-up are essential to ensure long-term success.

A limitation of this case report is the relatively short follow-up period, of six months, as the patient relocated abroad during the observation period, precluding long-term clinical evaluation of appliance retention, periodontal health, caries recurrence, occlusal changes, and eruption guidance.

## Conclusions

This case report highlights the importance of early diagnosis and timely intervention in pediatric patients with complex medical conditions to avoid further havoc for the child. The modified Groper appliance proved to be a simple and effective treatment option, providing restoration of esthetics and supporting speech, mastication, and occlusal function while serving as a functional space maintainer during the six-month follow-up period. The patient demonstrated satisfactory appliance retention and favorable clinical outcomes throughout this period. However, the relatively short follow-up duration remains a limitation of this report, and longer-term clinical evaluation is necessary to determine the appliance's effectiveness in maintaining space and supporting oral function throughout growth and dental development.

Furthermore, this case emphasizes the importance of a multidisciplinary approach involving close collaboration between pediatric dentists and pediatric nephrologists. Such coordinated care is essential for addressing both the immediate oral health needs and the long-term dental and craniofacial development of medically compromised children.

## References

[1] Saini N, Pradhan D, Tiwari S, Thakur R, Jain N (2024). Innovative aesthetic and functional rehabilitation in a two-and-a-half-year-old using a modified Groper's appliance. Cureus.

[2] Jabin Z, Verma SD, Agarwal N, Anand A, Waikhom N (2022). Groper's appliance - for esthetic rehabilitation of anterior missing teeth: two case reports. J Interdiscip Dent.

[3] Shanmugaavel AK, Gurunathan D, Sundararajan L (2016). Smile re-construction for the preschoolers using GRASCE appliance-two case reports. J Clin Diagn Res.

[4] Nadelman P, Bedran N, Magno MB, Masterson D, de Castro AC, Maia LC (2020). Premature loss of primary anterior teeth and its consequences to primary dental arch and speech pattern: a systematic review and meta-analysis. Int J Paediatr Dent.

[5] Wei SH (1988). Pediatric Dentistry: Total Patient Care. Febiger.

[6] Padubidri M, Pawar N, Padmawar N, Nara A, Joshi S, Mopagar V (2018). Dental management of patients with nephrotic syndrome- a report of 2 cases. Pravara Med Rev.

[7] Waggoner WF, Kupietzky A (2001). Anterior esthetic fixed appliances for the preschooler: considerations and a technique for placement. Pediatr Dent.

[8] Arikan V, Ocal D, Akcay M, Altug AT, Ozer L, Cesur E (2020). Growth-enabling modified Groper appliance for the replacement of permanent maxillary incisors: report of two cases. Australasian Orthodont J.

[9] Shrivastava U, Masih U, Chand BR, Manker A, Barjatya K, Juneja P (2021). Esthetic rehabilitation with modified Groper’s appliance in a four year old child: a case report. Int J Oral Health Dent.

[10] Joshi A, Anand R, Sanjana Kodical, Ishani Ratnaparkhi, Balasubramanian N, Sanghavi M (2021). Esthetic rehabilitation using a modified Groper’s appliance for a 3-year-old child: a unique case report. Int J Curr Res Rev.

[11] Jabin Z, Dudeja P, Dudeja K (2021). Management of bilateral dental agenesis with aesthetic rehabilitation by Groper's appliance - a six-year follow-up. J Clin Diagn Res.

[12] Anand S, Singh A, Jyoti D, Sulekha Sulekha, Seal B (2021). An esthetic approach for premature missing maxillary primary anterior teeth. Indian J Dent Sci.

[13] Georgakopoulou EA, Achtari MD, Afentoulide N (2011). Dental management of patients before and after renal transplantation. Stomatologija.

[14] Ghaly M, Hamila NA (2025). Evaluation of anterior fixed appliances on maxillary arch growth in preschool children. Sci Rep.

[15] Seth MP, Rani ST, Merum K, Raju S (2018). Fix smile for a while: an esthetic rehabilitation with Groper’s appliance - two case reports. Indian J Dent Adv.

